# Predicting stage B2 myxomatous mitral valve disease in dogs using machine learning and routine clinical data

**DOI:** 10.3389/fvets.2026.1924412

**Published:** 2026-09-11

**Authors:** Hasuk Nam, Kyungchang Jeong, Hanbit Seo, Yeon Chae, Byeong-Teck Kang, Euijong Lee, Taesik Yun, Hakhyun Kim

**Affiliations:** 1 Laboratory of Veterinary Internal Medicine, College of Veterinary Medicine, Chungbuk National University, Cheongju, Chungbuk, Republic of Korea; 2 School of Computer Science, Chungbuk National University, Cheongju, Chungbuk, Republic of Korea

**Keywords:** canine, diagnosis, gradient boosting, mitral valve disease, pimobendan

## Abstract

Early identification of Stage B2 myxomatous mitral valve disease in dogs is critical for initiating appropriate treatment to delay the onset of heart failure. Although echocardiography is the gold standard for diagnosing Stage B2 cardiac remodeling, it has limitations in general clinical settings owing to the limited availability of specialized equipment and the need for advanced technical expertise. We developed a machine learning model using routine clinical data to identify dogs with Stage B2 disease without echocardiography. A total of 387 client-owned dogs (252 non-Stage B2 dogs and 135 Stage B2 dogs) were evaluated. A gradient boosting algorithm was trained on 80% of the data and validated on a test dataset comprising the remaining 20%, incorporating demographic, hematological, serum biochemical, urinalysis, and thoracic radiographic variables. The model achieved an accuracy of 0.825 [95% confidence interval (CI): 0.738–0.900], a sensitivity of 0.815 (95% CI: 0.650–0.957), a specificity of 0.830 (95% CI: 0.729–0.919), and an area under the receiver operating characteristic curve of 0.922 (95% CI: 0.860–0.971). These findings demonstrate that machine learning can accurately classify Stage B2 status using routine clinical data, thereby assisting veterinary clinicians in the early detection and specialist referral when echocardiography is unavailable.

## 1 Introduction

Myxomatous mitral valve disease (MMVD) is the most common acquired cardiac disease in dogs, particularly in small breeds (1). It is a chronic and progressive condition characterized by degenerative changes in the mitral valve, leading to mitral regurgitation and the eventual development of congestive heart failure (CHF), which can be life-threatening in dogs (2).

The clinical staging of MMVD is based on the American College of Veterinary Internal Medicine (ACVIM) consensus guidelines, in which dogs are classified according to disease severity (3). Among these stages, distinguishing Stage B1 from Stage B2 is essential because Stage B2 represents dogs with significant structural cardiac enlargement, whereas dogs in Stage B1 may exhibit mild cardiac changes but do not meet Stage B2 criteria. Furthermore, the EPIC study demonstrated that the administration of pimobendan to dogs with significant left atrial and left ventricular enlargement, as defined by enrollment criteria that subsequently shaped the definition of ACVIM Stage B2 MMVD, significantly delayed the onset of CHF (4). Therefore, identifying dogs with ACVIM Stage B2 MMVD is important for delaying disease progression through the administration of pimobendan.

Although echocardiography is the gold standard diagnostic tool for assessing structural cardiac remodeling, which is critical for identifying ACVIM Stage B2, its practical application is often limited by the need for specialized equipment and advanced technical expertise (3). In this context, several studies have evaluated non-echocardiographic indicators of cardiac enlargement or disease severity in dogs with MMVD. These include complete blood count-derived inflammatory indices, such as the neutrophil-to-lymphocyte ratio; murmur intensity; radiographic indices, such as vertebral left atrial size (VLAS); and cardiac biomarkers (5–9). However, these approaches have generally focused on individual variables. Machine learning, a branch of artificial intelligence, can simultaneously process multiple variables and identify complex non-linear relationships within clinical data (10, 11).

Based on these characteristics, machine learning has been applied to the diagnosis and prognosis of various diseases in human and veterinary medicine (10–12). In veterinary medicine, previous studies have used routinely available clinical data, including signalment, clinical signs, and laboratory test results, to support the diagnosis or prediction of diseases such as hypercortisolism, hypoadrenocorticism, and chronic kidney disease (13–15).

Machine learning has also been applied in MMVD-related research, including clinical assessment, auscultation analysis, and radiographic image-based ACVIM classification (16–18). A previous study also developed a machine learning model for MMVD staging; however, the model incorporated echocardiographic parameters (19). Therefore, the application of machine learning to identify ACVIM Stage B2 using routinely available clinical data without echocardiographic parameters remains limited.

Based on these considerations, we hypothesized that a machine learning model integrating multiple non-echocardiographic clinical variables could accurately identify dogs with ACVIM Stage B2. This study aimed to develop a machine-learning-based model to identify ACVIM Stage B2 in dogs with MMVD using routinely available clinical data, including physical examination findings, thoracic radiographic measurements, and laboratory test results, while excluding echocardiographic parameters. By identifying dogs likely to have MMVD Stage B2, the model was intended to support clinical decision-making and guide further cardiac evaluation, including echocardiography.

## 2 Materials and methods

### 2.1 Study population

A search was conducted using the electronic medical records of dogs evaluated at Chungbuk National University Veterinary Teaching Hospital between January 2018 and December 2025 using two separate search queries for patient screening.

In the MMVD Stage B cohort, dogs previously diagnosed with Stage B MMVD were initially identified. Dogs were excluded from this cohort if they met any of the following criteria: (a) incomplete parameters for Stage B2 classification, specifically the grade of heart murmur, vertebral heart scale (VHS), left atrium-to-aorta ratio (LA/Ao), or normalized left ventricular internal diameter in diastole (LVIDdN) (3); (b) a history of prior use of cardiovascular medications, including pimobendan or diuretics, before presentation; or (c) the presence of other concurrent cardiac diseases, such as significant tricuspid valve disease, pulmonary hypertension, dilated cardiomyopathy or congenital cardiac disease. Stage B dogs were classified as Stage B1 or Stage B2 according to the 2019 ACVIM consensus guidelines (3). Stage B2 was defined by the presence of all the following criteria: heart murmur grade ≥ 3/6, VHS > 10.5, LA/Ao ≥ 1.6, and LVIDdN ≥ 1.7. Dogs were classified as Stage B1 if they did not meet the criteria for Stage B2.

In the healthy control cohort, dogs that underwent health examinations were initially identified based on electronic medical records. From these control candidates, subjects were excluded based on the following criteria: (a) incomplete thoracic radiographic records precluding VHS measurement; (b) detection of an audible heart murmur during physical examination; (c) prior use of cardiovascular medications, including pimobendan or diuretics; and (d) clinical or laboratory evidence of underlying systemic disease. The remaining dogs were defined as eligible healthy controls.

For machine learning model development, the study population was classified into two distinct groups: B2 and non-B2. The B2 group comprised dogs with confirmed MMVD Stage B2. In contrast, the non-B2 group was established by pooling dogs classified as having MMVD Stage B1 and healthy controls.

### 2.2 Clinical and diagnostic evaluations

Baseline clinical and clinicopathological data for all enrolled dogs at the time of the initial evaluation were obtained from the electronic medical records. These data included findings from a thorough physical examination, including heart murmur grading using a 6-point scale, and a complete blood count performed using an automated hematology analyzer (ProCyte Dx Analyzer, IDEXX Laboratories, Westbrook, ME, USA). Serum biochemical profiles were evaluated using an automated biochemical analyzer (Hitachi 7020, Hitachi High-Technologies Co., Tokyo, Japan). Comprehensive urinalysis results, including urine specific gravity (USG) (PAL-USG; ATAGO Co., Ltd., Tokyo, Japan) and the urine protein-to-creatinine ratio (Catalyst One; IDEXX Laboratories, Westbrook, ME, USA), were also reviewed. In addition, thoracic radiographs obtained in right lateral recumbency were evaluated to assess VHS and VLAS according to previously described methods (8, 20).

### 2.3 Echocardiography

To evaluate the ACVIM classification criteria in dogs with Stage B MMVD, echocardiographic records of LA/Ao and LVIDdN were reviewed to establish the definitive ACVIM stage. Echocardiographic examinations were performed using commercial ultrasound systems, including the Aloka Prosound Alpha 7 (Wallingford, CT, USA) and Philips EPIQ7 (Bothell, WA, USA). Right parasternal short-axis echocardiography was used to measure LA/Ao, and M-mode echocardiography was used to assess the left ventricular internal dimension at end-diastole (LVIDd) for the calculation of LVIDdN (21–24). All parameters were averaged from two consecutive measurements. These echocardiographic parameters were used for staging and were excluded from machine learning model training.

### 2.4 Machine learning

The predictive machine learning model incorporated routine clinical variables as input features, as shown in Table 1. To handle partially missing laboratory values, missing data were imputed using the k-nearest neighbors (k-NN) algorithm, and the features were subsequently scaled during preprocessing (25). The dataset was randomly divided into an 80% training set and a 20% test set to develop and validate the model. To assess model stability against stochastic variation, the same training and evaluation procedure was repeated across 20 independent random seeds, with a new random 80:20 train-test split generated for each run.

**Table 1:** Routine clinical variables used as input features for the predictive machine learning model.

Physical examination	CBC	Serum chemistry	Urinalysis	Thoracic radiographic measurements
Age (years)	White blood cell (x 10^3^/μl)	Total protein (g/dl)	USG	VHS
Sex	Monocyte count (x 10^3^/μl)	Albumin (g/dl)	Urine protein–to–creatinine ratio	VLAS
Breed	Lymphocyte count (x 10^3^/μl)	Globulin (g/dl)		
Body weight (kg)	Neutrophil count (x 10^3^/μl)	Alanine aminotransferase (IU/L)		
Body condition score (9–point scale)	Eosinophil count (x 10^3^/μl)	Alkaline phosphatase (IU/L)		
Heart rate (bpm)	Basophil count (x 10^3^/μl)	Total bilirubin (mg/dl)		
Respiratory rate (rpm)	Packed cell volume (%)	Blood urea nitrogen (mg/dl)		
Systolic blood pressure (mmHg)	Hemoglobin (g/dl)	Creatinine (mg/dl)		
Murmur grade (6–point scale)	Mean corpuscular volume (fL)	Symmetric dimethylarginine (μg/dl)		
	Mean corpuscular hemoglobin (pg)	Total cholesterol (mg/dl)		
	Mean corpuscular hemoglobin concentration (g/dl)	Triglycerides (mg/dl)		
	Platelets (x 10^3^/μl)	Glucose (mg/dl)		
	Platelet–to–lymphocyte ratio	Lactate (mmol/L)		
	Neutrophil–to–lymphocyte ratio	C–reactive protein (mg/L)		
		Sodium (mmol/L)		
		Potassium (mmol/L)		
		Chloride (mmol/L)		
		Calcium (mg/dl)		
		Phosphorus (mg/dl)		
		Magnesium (mg/dl)		

USG, urine specific gravity; VHS, vertebral heart scale; VLAS, vertebral left atrial size.

A gradient boosting machine was implemented using scikit-learn's GradientBoostingClassifier (15, 26, 27). Hyperparameter optimization was performed using a Bayesian approach combined with five-fold cross-validation to prevent overfitting. The final optimized model consisted of 100 decision trees with a learning rate of 0.03, a maximum tree depth of 5, a minimum of two samples per leaf, a subsampling fraction of 0.8, and log2 feature subsampling.

Subsequently, predictor importance was evaluated to determine the relative contribution of each variable to the final optimized model. The resulting importance scores were normalized so that their total sum across all features was 1.0, representing the relative contribution of each variable. Based on the feature importance ranking, two reduced models were additionally evaluated using the same modeling procedure: one incorporating the three highest-ranked variables (VHS, VLAS, and C-reactive protein [CRP]) and another incorporating two radiographic variables (VHS and VLAS). All machine learning procedures were performed using Python and the scikit-learn library.

### 2.5 Statistical analyses

All statistical analyses were performed using commercially available software (Prism 11; GraphPad Software Inc., San Diego, CA, USA). Data normality was assessed using the Shapiro-Wilk test. Because the data were not normally distributed, continuous variables were expressed as medians and interquartile ranges, and differences between the non-B2 and B2 groups were analyzed using the Mann-Whitney U test. Categorical variables were compared using the chi-squared test or Fisher's exact test. A *P* value < 0.05 was considered statistically significant for all analyses.

Model performance was evaluated in terms of discrimination and calibration. Discriminative performance was assessed using confusion matrix-derived metrics, including accuracy, sensitivity, specificity, and the area under the curve (AUC), all reported with 95% confidence intervals (CIs). The optimal decision threshold was determined using Youden's index from the receiver operating characteristic (ROC) curve analysis. Calibration of the predicted probabilities was assessed using a calibration plot and the Brier score. ROC curve analyses were performed using GraphPad Prism.

## 3 Results

### 3.1 Demographic and physical examination

**Figure 1:**
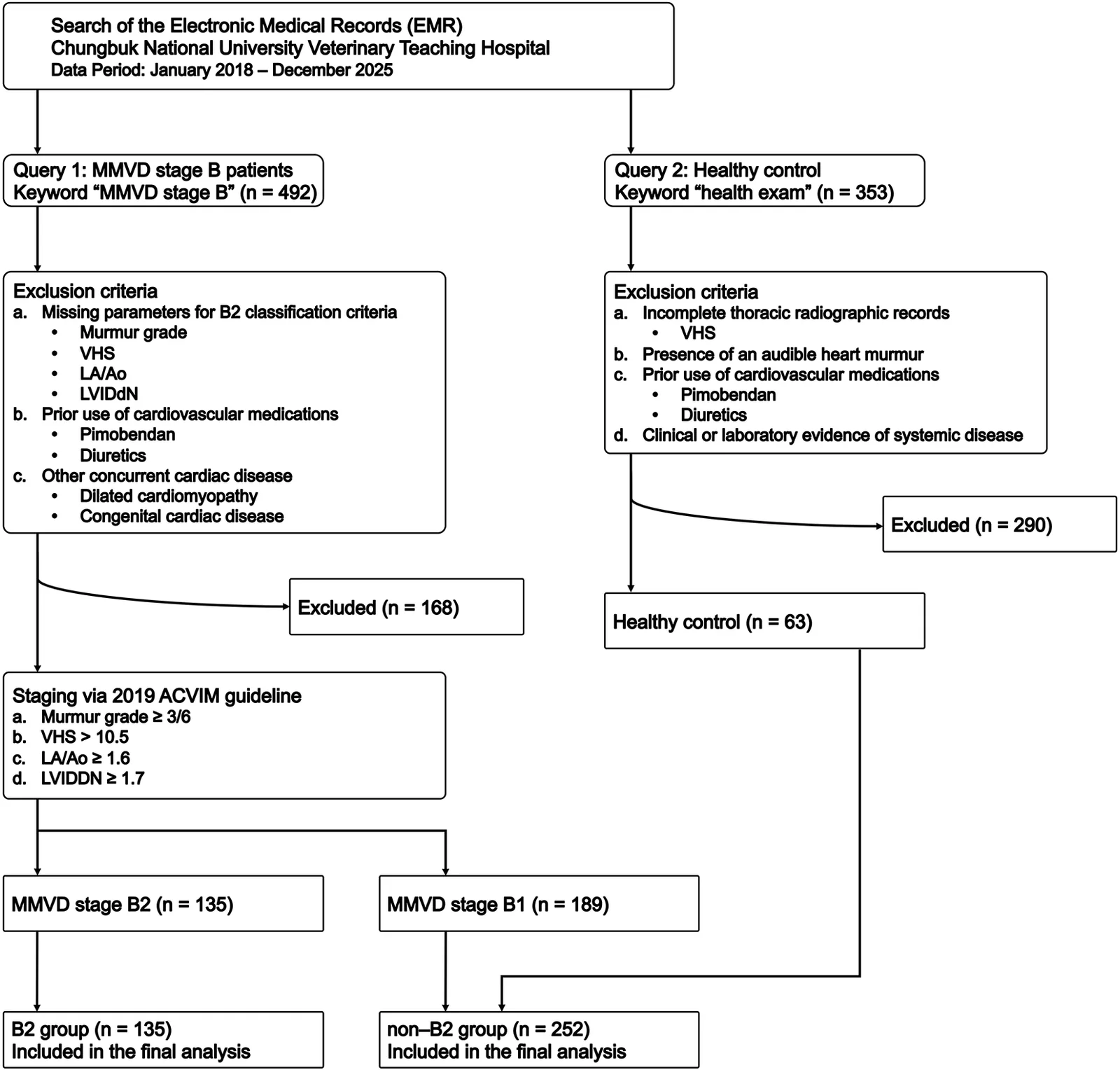
Flow chart of the study population. Electronic medical records from Chungbuk National University Veterinary Teaching Hospital between January 2018 and December 2025 were reviewed. Dogs were assigned to the B2 or non-B2 groups based on the 2019 American College of Veterinary Internal Medicine (ACVIM) staging guidelines. The non-B2 group included dogs with ACVIM Stage B1 disease and healthy controls.

Finally, 387 dogs were included in the final analysis (Figure 1). The B2 group consisted entirely of dogs with confirmed ACVIM Stage B2 disease (*n* = 135). The non-B2 group (*n* = 252) comprised eligible healthy controls (*n* = 63) and dogs with confirmed ACVIM Stage B1 disease (*n* = 189). Baseline clinical and physical examination characteristics are presented in Table 2. Statistically significant differences were observed between the two groups in terms of age (*P* < 0.0001), heart rate (*P* = 0.0041), and heart murmur grade (*P* < 0.0001). No statistically significant differences were observed in the other parameters listed in Table 2 (*P* > 0.05). A total of 23 and 14 different breeds were represented in the non-B2 and B2 groups, respectively, with Maltese, Pomeranian, and Shih Tzu being the most common breeds in both groups. The proportions of these major breeds showed no statistically significant differences between the two groups (*P* > 0.05; data not shown).

**Table 2:** Baseline characteristics of dogs included in the study.

Characteristics	Non–B2 group (*n* = 252)	B2 group (*n* = 135)	*P* value
Age (years)	9 (7–12)	11 (9–13)	< 0.0001^*^
Sex			0.3284
Male (neutered)	131 (117)	77 (66)	
Female (neutered)	121 (107)	58 (46)	
Body weight (kg)	4.40 (3.23–5.47)	4.35 (3.26–5.40)	0.9629
Body condition score			0.0884
Under ideal (< 4/9)	12 (4.76%)	14 (10.37%)	
Ideal (4–5/9)	167 (66.27%)	80 (59.26%)	
Over ideal (>5/9)	73 (28.97%)	41 (30.37%)	
Heart rate (bpm)	144 (124–162)	150 (132–176)	0.0041^*^
Respiratory rate (rpm)	30 (24–42)	30 (30–51)	0.1183
Systolic blood pressure (mmHg)	140 (124–150)	140 (120–150)	0.9142
Heart murmur grade	3 (0–4)	4 (4–4)	< 0.0001^*^

Continuous variables are presented as medians (interquartile ranges). Categorical variables are presented as the number of dogs (%), except for sex, which is presented as the total number (number neutered). Asterisks indicate statistically significant differences between the non–B2 and B2 groups (*P* < 0.05).

### 3.2 Clinicopathological and radiographic data

The hematological, serum biochemical, urinalysis, and thoracic radiographic results are presented in Table 3. Statistically significant differences between the non-B2 and B2 groups were observed in basophil count (*P* = 0.0128), packed cell volume (*P* = 0.0017), hemoglobin (*P* = 0.0011), mean corpuscular volume (*P* = 0.0001), mean corpuscular hemoglobin (MCH) (*P* < 0.0001), blood urea nitrogen (BUN) (*P* = 0.0136), USG (*P* = 0.0027), VHS (*P* < 0.0001), and VLAS (*P* < 0.0001). No significant differences were observed in the other clinicopathological parameters.

**Table 3:** Clinical, laboratory, and radiographic characteristics of dogs included in the study.

Characteristics	Non–B2 group	B2 group	*P* value
White blood cell (x 10^3^/μl)	9.18 (7.49–11.70)	9.11 (7.43–11.19)	0.5857
Monocyte count (x 10^3^/μl)	0.50 (0.36–0.65)	0.54 (0.36–0.79)	0.7174
Lymphocyte count (x 10^3^/μl)	1.92 (1.52–2.38)	1.88 (1.53–2.27)	0.0644
Neutrophil count (x 10^3^/μl)	6.28 (4.70–8.27)	5.89 (4.82–7.74)	0.4259
Eosinophil count (x 10^3^/μl)	0.35 (0.23–0.50)	0.33 (0.22–0.47)	0.4796
Basophil count (x 10^3^/μl)	0.02 (0.01–0.05)	0.02 (0.01–0.03)	0.0128^*^
Packed cell volume (%)	46.9 (41.3–51.5)	44.0 (39.7–49.3)	0.0017^*^
Hemoglobin (g/dl)	16.7 (14.8–17.9)	15.7 (14.2–17.3)	0.0011^*^
Mean corpuscular volume (fL)	64.8 (62.3–66.7)	62.9 (60.8–65.4)	0.0001^*^
Mean corpuscular hemoglobin (pg)	22.9 (22.1–23.6)	22.4 (21.4–23.0)	< 0.0001^*^
Mean corpuscular hemoglobin concentration (g/dl)	35.4 (34.6–36.1)	35.4 (35.0–35.9)	0.7588
Platelets (x 10^3^/μl)	384 (295–483)	382 (293–473)	0.7376
Platelet–to–lymphocyte ratio	205.6 (140.4–265.7)	200.7 (130.5–280.9)	0.9447
Neutrophil–to–lymphocyte ratio	3.3 (2.31–4.46)	3.27 (2.63–4.26)	0.7315
Total protein (g/dl)	6.3 (6.1–6.7)	6.4 (5.9–6.8)	0.8854
Albumin (g/dl)	3 (2.7–3.3)	3.1 (2.8–3.3)	0.1499
Globulin (g/dl)	3.4 (2.9–3.7)	3.2 (2.9–3.6)	0.2997
Alanine aminotransferase (IU/L)	50 (38–74)	48 (33–86)	0.8568
Alkaline phosphatase (IU/L)	169 (86–388)	187 (99–477)	0.1843
Total bilirubin (mg/dl)	0.01 (0–0.1)	0 (0–0.1)	0.1867
Blood urea nitrogen (mg/dl)	19.2 (14.9–25.7)	21.1 (17.1–28.9)	0.0136^*^
Creatinine (mg/dl)	0.89 (0.70–1.07)	0.90 (0.70–1.00)	0.9943
Symmetric dimethylarginine (μg/dl)	9 (7–12)	9 (7–12)	0.9133
Total cholesterol (mg/dl)	227 (180–277)	206 (180–264)	0.3285
Triglycerides (mg/dl)	71 (45–140)	71 (51–107)	0.8882
Glucose (mg/dl)	109 (102–122)	111 (100–124)	0.7772
Lactate (mmol/L)	2.38 (1.80–3.38)	2.40 (1.84–3.09)	0.7002
C–reactive protein (mg/L)	5.43 (5.00–10.02)	6.50 (5.00–11.41)	0.1200
Sodium (mmol/L)	147 (144–148)	147 (144–149)	0.5994
Potassium (mmol/L)	4.8 (4.5–5.1)	4.7 (4.3–5.0)	0.4753
Chloride (mmol/L)	114 (111–116)	114 (112–116)	0.4961
Calcium (mg/dl)	9.7 (9.2–10.2)	9.9 (9.2–10.3)	0.1117
Phosphorus (mg/dl)	3.5 (3.0–4.1)	3.5 (3.0–4.0)	0.9768
Magnesium (mg/dl)	2.0 (1.8–2.2)	2.0 (1.8–2.2)	0.5605
USG	1.030 (1.021–1.041)	1.025 (1.016–1.033)	0.0027^*^
Urine protein–to–creatinine ratio	0.20 (0.10–0.25)	0.20 (0.09–0.29)	0.6654
VHS	10.2 (9.7–10.68)	11.2 (10.70–11.90)	< 0.0001^*^
VLAS	2.1 (2.0–2.3)	2.6 (2.4–2.9)	< 0.0001^*^

Continuous variables are presented as medians (interquartile ranges). Asterisks indicate statistically significant differences between the non–B2 and B2 groups (*P* < 0.05). USG, urine specific gravity; VHS, vertebral heart scale; VLAS, vertebral left atrial size.

### 3.3 Machine learning model performance

The total study population (*n* = 387) was randomly divided, with 80% of the dogs (*n* = 307) assigned to the training cohort and the remaining 20% (*n* = 80) assigned to the test cohort. Using routine clinicopathological variables, a classification model was developed using a gradient boosting algorithm. Based on the test dataset evaluation, the model achieved an accuracy of 0.825 (95% CI: 0.738–0.900), a sensitivity of 0.815 (95% CI: 0.650–0.957), a specificity of 0.830 (95% CI: 0.729–0.919), and an AUC of 0.922 (95% CI: 0.860–0.971).

**Figure 2:**
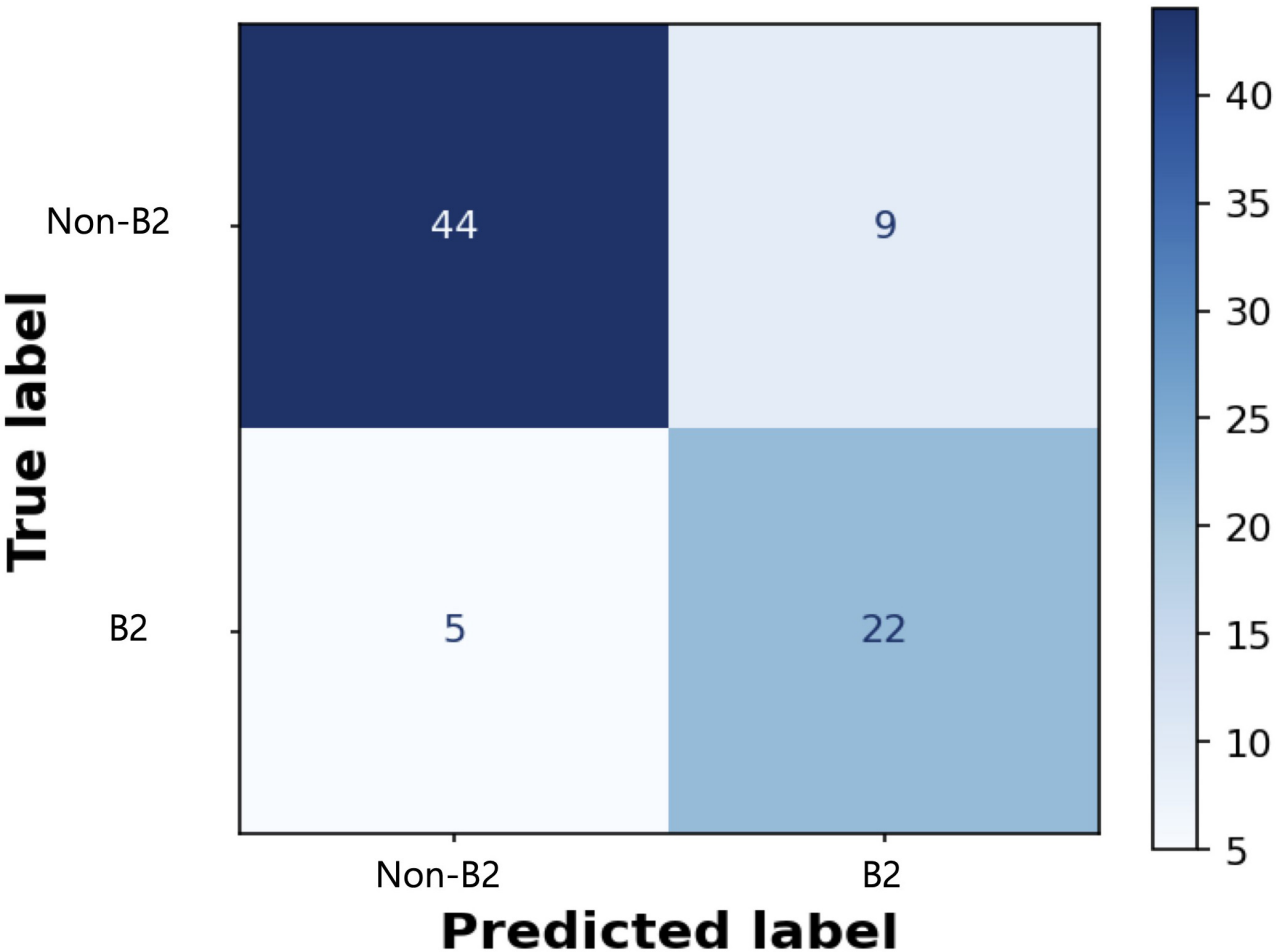
Confusion matrix of the machine learning model for detecting American College of Veterinary Internal Medicine Stage B2 myxomatous mitral valve disease. This figure shows the distribution of the true and predicted labels in the test dataset. The model correctly classified 44 of the 53 non-B2 dogs and 22 of the 27 B2 dogs. The color bar on the right indicates the number of dogs corresponding to the color intensity of the cells, with darker blue representing a higher number of dogs.

**Figure 3:**
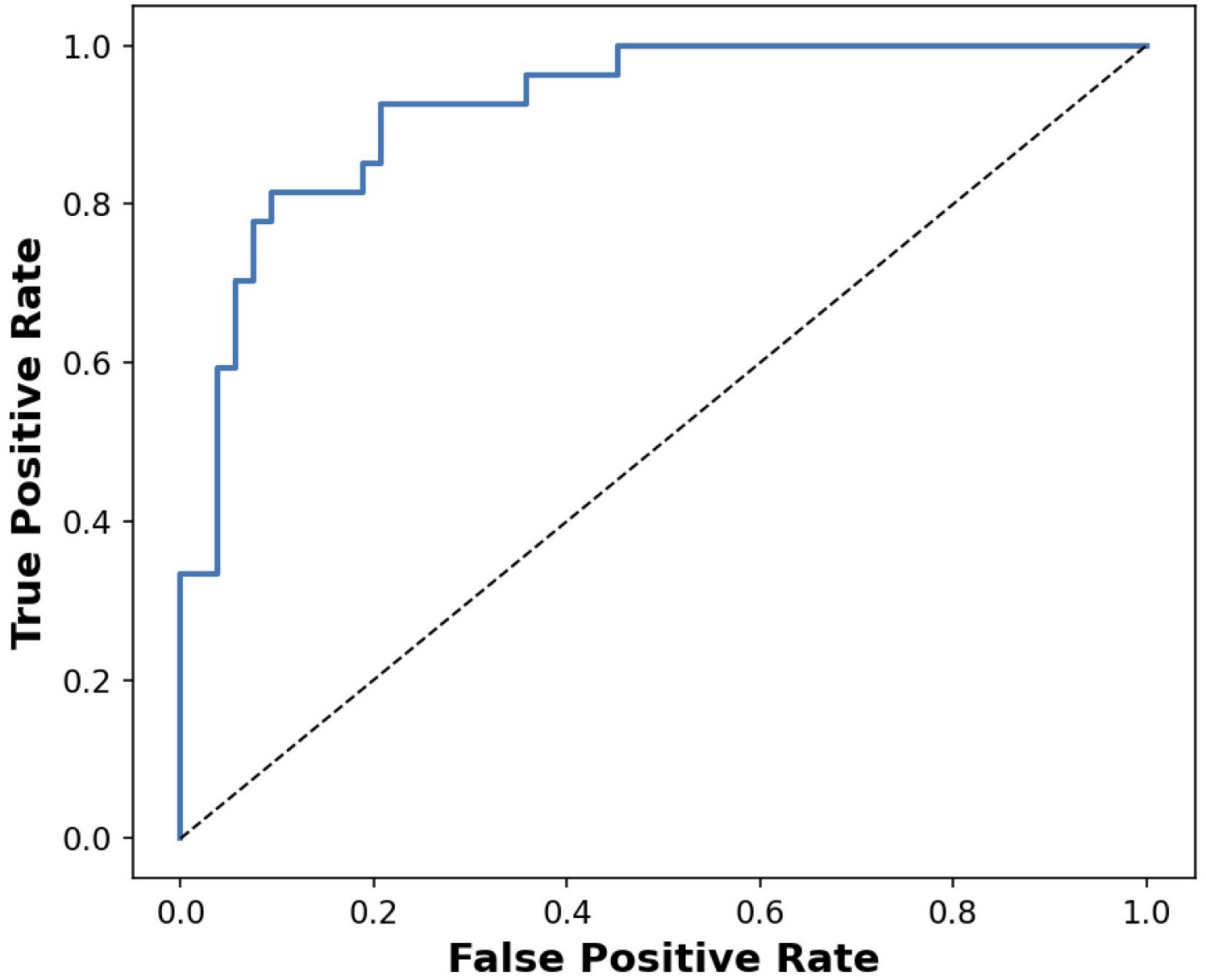
Receiver operating characteristic curve of the final machine learning model for detecting American College of Veterinary Internal Medicine Stage B2 myxomatous mitral valve disease. The curve illustrates the diagnostic performance of the model on the test dataset. The area under the receiver operating characteristic curve was 0.922.

The resulting classification results are presented in the confusion matrix (Figure 2). The algorithm correctly identified 44 of the 53 non-B2 dogs (83.0%) and 22 of the 27 B2 dogs (81.5%). In addition, the discriminative performance of the model across different decision thresholds is illustrated by the ROC curve shown in Figure 3.

**Figure 4:**
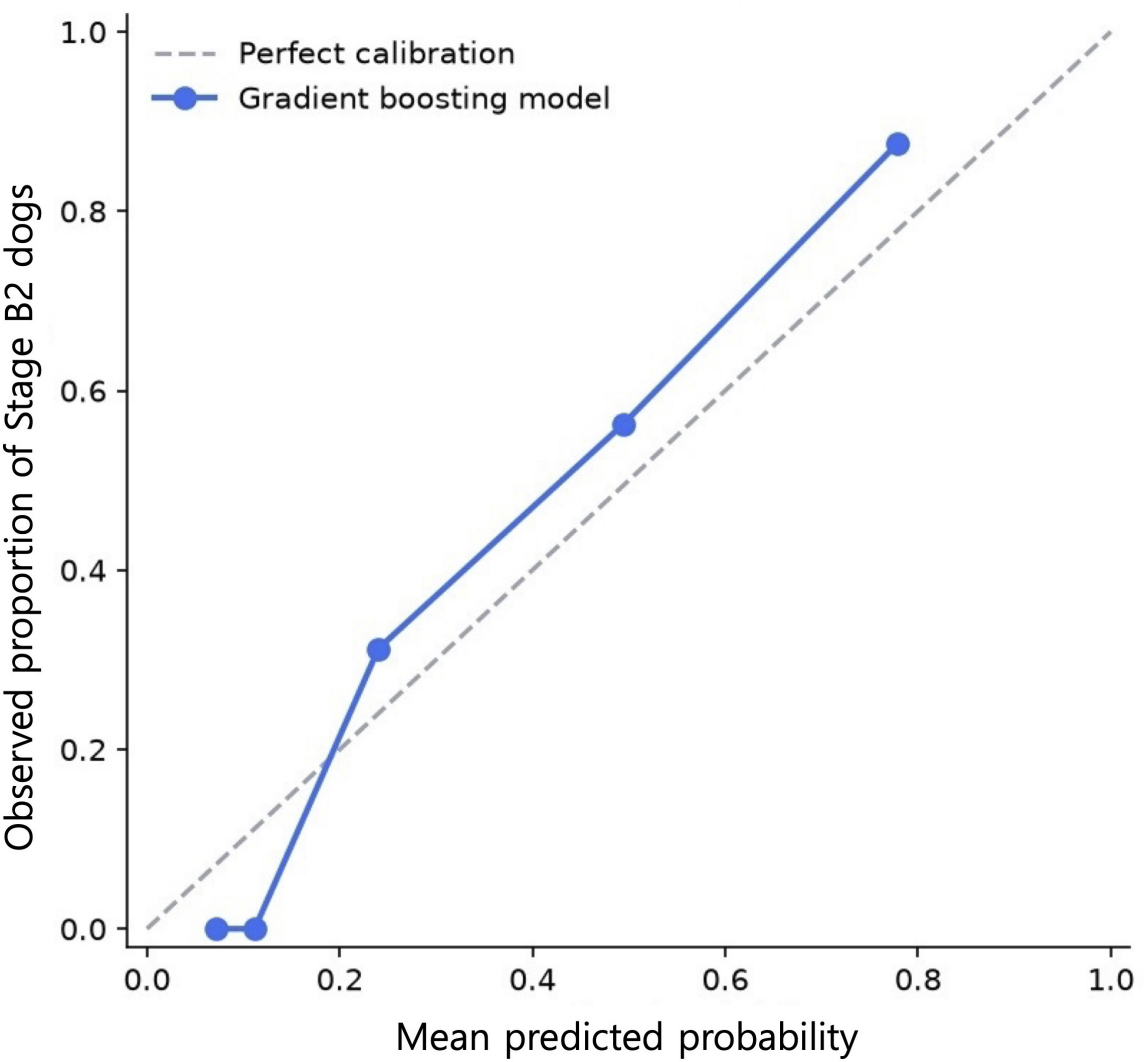
Calibration plot of the gradient boosting model for detecting American College of Veterinary Internal Medicine Stage B2 myxomatous mitral valve disease. The calibration plot compares the mean predicted probability with the observed proportion of Stage B2 dogs in the test dataset (*n* = 80). Predicted probabilities were grouped into five bins, with each data point representing 16 dogs (*n* = 16). The dashed diagonal line represents perfect calibration. The Brier score was 0.120.

The calibration plot showed generally reasonable agreement between the predicted probabilities and the observed proportion of Stage B2 dogs, with some deviation from perfect calibration across the probability bins (Figure 4). The Brier score was 0.120.

To further assess the stability of model performance across random data splits, the training and evaluation process was repeated across 20 independent random seeds, with a new random 80:20 train-test split generated for each run. Across the 20 runs, the AUC was 0.910 ± 0.008 (mean ± standard deviation; range, 0.894–0.926), while accuracy, sensitivity, and specificity were 0.835 ± 0.024, 0.694 ± 0.037, and 0.909 ± 0.029, respectively.

### 3.4 Feature importance analysis and reduced-variable model performance

**Figure 5:**
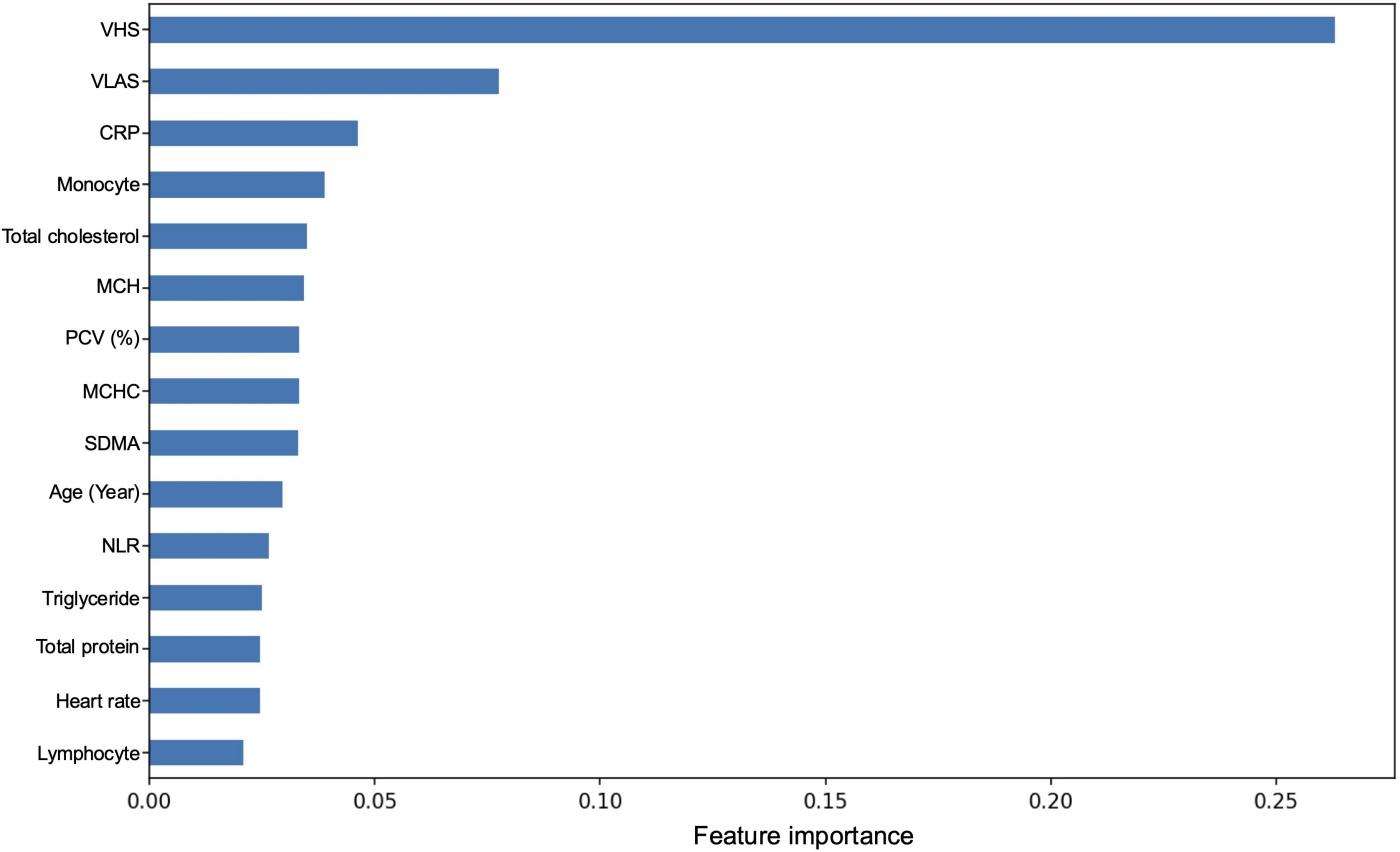
Feature importance ranking of the gradient boosting model. The horizontal bars represent the relative importance scores of the top 15 variables. Importance scores were normalized so that their sum across all evaluated features was 1.0. Vertebral heart scale had the highest importance score (0.263), followed by vertebral left atrial size (0.078), C-reactive protein (0.046), and monocyte count (0.039). VHS, vertebral heart scale; VLAS, vertebral left atrial size; CRP, C-reactive protein; MCH, mean corpuscular hemoglobin; PCV, packed cell volume; MCHC, mean corpuscular hemoglobin concentration; SDMA, symmetric dimethylarginine; NLR, neutrophil-to-lymphocyte ratio.

The feature importance ranking demonstrates the relative contribution of each variable to the predictive performance of the final gradient boosting model, with the top 15 variables among all evaluated parameters shown in Figure 5. Among these variables, VHS had the highest relative importance score (0.263), followed by VLAS (0.078), CRP (0.046), monocyte count (0.039), total cholesterol (0.035), MCH (0.034), and packed cell volume (0.033).

Based on the feature importance ranking, additional models using only the three highest-ranked variables (VHS, VLAS, and CRP) and the two radiographic variables (VHS and VLAS) were evaluated. The model using VHS, VLAS, and CRP achieved an accuracy of 0.769, a sensitivity of 0.667, a specificity of 0.824, and an AUC of 0.864. The model using VHS and VLAS alone achieved an accuracy of 0.756, a sensitivity of 0.667, a specificity of 0.804, and an AUC of 0.838.

## 4 Discussion

The present study evaluated whether ACVIM Stage B2 in dogs with MMVD could be identified using a machine learning algorithm based on routinely available clinical data obtained in clinical practice without echocardiographic variables. The gradient boosting model showed good discriminatory performance on the test dataset, with an accuracy of 0.825, a sensitivity of 0.815, a specificity of 0.830, and an AUC of 0.922. These results indicate that routinely obtained clinical data, including physical examination findings, laboratory results, and thoracic radiographic measurements, may provide sufficient information to support the identification of dogs with a high probability of MMVD Stage B2. Across 20 additional runs with different random train-test partitions, discrimination remained stable, with a mean AUC of 0.910 ± 0.008. Accuracy and specificity also showed relatively limited variation, whereas sensitivity showed greater variability, with a mean of 0.694 ± 0.037. These findings suggest that the overall discriminatory ability of the model was relatively stable across different data partitions, although sensitivity to Stage B2 cases showed greater variation. This variability may reflect the relatively small number of Stage B2 dogs included in each test set. In addition to discrimination, the model showed generally reasonable calibration on the test dataset, with a Brier score of 0.120. The calibration plot showed overall agreement between predicted probabilities and observed outcomes, although some deviation from perfect calibration was observed across the probability bins.

As previously described, pimobendan administration to dogs meeting the significant cardiac enlargement criteria that subsequently shaped the definition of ACVIM Stage B2 MMVD can delay progression to CHF (4). Therefore, the detection of Stage B2 is clinically important because this stage is characterized by significant structural cardiac remodeling, whereas dogs in Stage B1 do not meet these criteria (3). Echocardiography remains the “gold standard” for evaluating cardiac remodeling and determining the ACVIM stage in dogs with MMVD. However, it requires specialized equipment and technical expertise and may not be readily available (3, 18). Previous studies have also reported variability in left atrial size measurements among experienced examiners (28). These limitations support the clinical relevance of identifying MMVD Stage B2 without echocardiographic variables in general practice.

To overcome these limitations, alternative diagnostic approaches and advanced analytical methods for MMVD staging have been investigated. For instance, neutrophil-to-lymphocyte and monocyte-to-lymphocyte ratios have been proposed as inflammatory biomarkers associated with MMVD severity; however, these ratios showed limited ability to distinguish early cardiac remodeling between dogs with ACVIM Stages B1 and B2 (5). Similarly, although heart murmur intensity is associated with the probability of CHF, its ability to predict Stage B2 remains limited, with approximately 56% of dogs with moderate murmurs and 29% with loud murmurs lacking evidence of cardiac remodeling (7). NT-proBNP is recognized as a useful biomarker of cardiac remodeling (9). However, it was measured in only 15 of 135 dogs in the B2 group and 12 of 252 dogs in the non-B2 group. Given the substantial proportion of missing data, NT-proBNP could not be incorporated into the predictive model. Furthermore, traditional statistical methods often evaluate selected variables individually or within predefined models, whereas machine learning can incorporate multiple clinical variables and model their complex relationships (10). Recent veterinary research has applied machine learning models to evaluate MMVD severity and classify disease stages by incorporating various clinical and echocardiographic indices (19).

In contrast to previous approaches that relied on advanced imaging, the present study developed a machine learning model integrating routine clinical data without echocardiographic indices for the detection of MMVD Stage B2 in dogs. Physical examination variables, including age, heart rate, and heart murmur grade, differed significantly between the B2 and non-B2 groups. Differences in age and heart murmur grade between the B2 and non-B2 groups are clinically plausible because MMVD is a chronic and progressive disease, and more advanced disease is generally associated with older age and a more prominent cardiac murmur. The difference in heart rate is also consistent with a previous machine learning-based study on MMVD classification, although heart rate should be interpreted cautiously because it can be influenced by physical activity, stress, and the clinical environment (16).

Several laboratory results, including basophil count, erythrocyte-related indices, BUN, and USG, also showed significant differences between the groups. Although the basophil count differed significantly, the absolute values were low in both groups, and their clinical relevance remains unclear. Therefore, this finding may represent a nonspecific hematological difference rather than a direct marker of cardiac remodeling. Other laboratory parameters are also not direct indicators of cardiac remodeling but may reflect secondary systemic changes accompanying MMVD progression. The alterations in BUN and USG levels may reflect age-related differences in renal function or other systemic factors, as the B2 group comprised significantly older dogs than the non-B2 group (29). Regarding the erythrocyte profile, a decreased packed cell volume is consistent with advanced stages of heart disease (30). Although PCV, hemoglobin, mean corpuscular volume, and MCH differed significantly between the groups, the magnitude of these differences was modest, and their clinical relevance remains unclear.

In the present study, VHS and VLAS were identified as the most important contributors to the model, with relative importance scores of 0.263 and 0.078, respectively (Figure 5). This is consistent with previous studies reporting these measurements as useful indicators of cardiac enlargement in dogs with MMVD (3, 4, 8).

However, the overall performance of the model cannot be fully explained by these radiographic variables alone because the model was developed using multiple routinely available clinical variables. This finding reflects the ability of the model to integrate multiple clinical variables rather than rely on fixed radiographic thresholds. The ACVIM guideline suggests that, in the absence of echocardiography, VHS ≥ 11.5 or VLAS ≥ 3.0 can support a presumptive diagnosis of Stage B2 (3). However, in the present study, the median VHS and VLAS values in the B2 group were 11.2 and 2.6, respectively, which were below the suggested cutoff values. This suggests that relying solely on fixed radiographic thresholds may lead to the underdiagnosis of dogs with Stage B2. To directly assess whether the performance of the model was attributable to the radiographic variables alone, an additional model using only VHS and VLAS was evaluated. This radiographic-only model achieved an AUC of 0.838, compared with 0.922 for the full model.

To further examine whether a simplified model incorporating the highest-ranked variables could retain predictive performance, CRP was added to the radiographic variables (VHS and VLAS). The resulting three-variable model achieved an AUC of 0.864, which was higher than that of the radiographic-only model (0.838) but remained lower than that of the full model. These findings suggest that CRP may provide additional discriminatory information beyond radiographic measurements, while a simplified model incorporating fewer clinical variables may warrant further investigation.

However, the proposed model is not intended to replace echocardiography in dogs with MMVD because echocardiography remains the gold standard for assessing cardiac remodeling indices (LA/Ao ratio and LVIDdN). Rather, the model may be most useful in clinical settings where echocardiography is not readily available, as a screening and decision-support tool to identify dogs requiring further echocardiographic evaluation and to help prioritize the urgency of referral. The model is intended to utilize routinely available clinical data when such information has already been obtained, rather than to require comprehensive laboratory testing solely for the purpose of determining the need for echocardiography.

This study has some limitations. First, the data were collected retrospectively from a single veterinary institution, which should be considered when interpreting the model performance. Additionally, although significant differences were observed between the non-B2 and B2 groups in several variables, including basophil count, BUN, USG, and erythrocyte-related indices, these variables were not direct indicators of cardiac remodeling. As discussed, these alterations may reflect age-related changes or secondary systemic complications associated with MMVD progression, rather than cardiac structural changes. Therefore, caution is required when interpreting these clinicopathological differences in a predictive context. Second, VHS and VLAS, the two most important predictors in the model, are both derived from thoracic radiographs. Accordingly, suboptimal image quality, patient positioning, or measurement variability may adversely affect model performance. Third, NT-proBNP was not included in our predictive model. Although NT-proBNP is a valuable biomarker for assessing MMVD severity, it was infrequently measured in our study cohort. Fourth, external validation using an independent dataset was not performed. Therefore, further validation using multicenter datasets is required. Finally, healthy controls and dogs with Stage B1 MMVD were combined into the non-B2 group to reflect the intended binary screening setting of the model, in which the likelihood of Stage B2 disease is assessed to support the decision for further echocardiographic evaluation. However, this grouping does not directly address the clinically important distinction between Stages B1 and B2. Therefore, future studies should evaluate this model specifically in dogs with ACVIM Stages B1 and B2 MMVD.

Despite these limitations, the gradient boosting model developed in this study identified dogs with a high probability of MMVD Stage B2 using routine clinical data without echocardiographic parameters. The model showed good discriminatory performance and complemented radiographic evaluation by integrating multiple clinical variables. Therefore, this model can serve as a decision-support tool to help clinicians identify dogs requiring further echocardiographic evaluation. Further training and external validation could improve the model's performance and clinical applicability.

## Data Availability

The original contributions presented in the study are included in the article, further inquiries can be directed to the corresponding authors.
